# The policy case for designating EMS teams for vulnerable patient populations: Evidence from an intervention in Boston

**DOI:** 10.1007/s10729-023-09635-6

**Published:** 2023-04-12

**Authors:** Mark Brennan, Sophia Dyer, Jonas Jonasson, James Salvia, Laura Segal, Erin Serino, Justin Steil

**Affiliations:** 1 Boston Emergency Medical Services, 785 Albany Street, Boston, MA 02118 USA; 2 Massachusetts Institute of Technology, 77 Massachusetts Avenue, Cambridge, MA 02134 USA

**Keywords:** Emergency medical services, Service segmentation, Analytics, Policy case, Equity, Homelessness, Addiction, Implementation

## Abstract

This study documents more than five years of analysis that drove the policy case, deployment, and retrospective evaluation for an innovative service model that enables Boston Emergency Medical Services (EMS) to respond quickly and effectively to investigation incidents in an area of heavy need in Boston. These investigation incidents are typically calls for service from passers-by or other third-party callers requesting that Boston EMS check in on individuals, often those who may appear to have an altered mental status or to be unhoused. First, this study reports the pre-intervention analytics in 2017 that built the policy case for service segmentation, a new Community Assistance Team designated “Squad 80” that primarily responds to investigation incidents in one broad area of the city with high rates of substance abuse and homelessness, helping patients who often refuse ambulance transport connect to social services. Second, this study reports a post-intervention, observational evaluation of its operational advantages and trade-offs. We observe that incidents involving the Community Assistance Team have significantly shorter response times and result in fewer transports to emergency departments than investigation incidents not involving the unit, leading to fewer ambulance unit-hours utilized across the system. This study documents the descriptive analytics that built the successful policy case for a substantive change in the healthcare-delivery supply chain in Boston and how this change offers operational advantages. It is written to be an accessible guide to the analysts and policy makers considering emergency services segmentation, an important frontier in equitable public-service delivery.

## Highlights

- There are active discussions on how to adapt emergency medical services to the complex needs of vulnerable urban populations—specifically unhoused patients and those facing substance-use disorders.
- This descriptive field study offers an operational perspective on a new emergency medical service model that uses service segmentation to better align resources with patient needs.
- The policy case for segmentation is derived from incidents with a high geographic concentration and that disproportionately involve vulnerable patients with a high need for social services and a low need for transport to a hospital.
- This evidence justified the creation of a specialized Community Assistance Team within Boston EMS, which we find has shorter response times and results in significantly fewer transports to emergency departments than comparable incidents not involving it.

## Introduction

Homelessness and substance use disorders are public health issues that emergency medical service systems are often asked to address as the healthcare safety net and as part of broader community response. In late 2017, Boston Emergency Medical Services (EMS) designed and deployed an innovative service model: a specialized team, called the Community Assistance Team, designated “Squad 80,” to respond to investigation incidents in central Boston. The categories of calls that we describe here as investigation incidents are: a report by a caller of a third-party medical event, categorized as an “unknown EMS”; a request by a caller for a medical investigation of a third-party’s well-being, categorized as an “EMS investigation”; or a request by a caller for medical assistance for a third-party, categorized as “request for EMS assistance.” These investigations generally arise when passers-by call 9–1-1 to request that emergency medical services check on individuals they have seen, often because those individuals are sleeping outside or appear to have an altered mental status.

The concept for Squad 80 arose from the recognition that investigation incidents have a low propensity for transport, but can still occupy an ambulance for an extended period of time, and are best served through linkage to city services. Patients can benefit from experienced emergency medical technicians (EMTs) with specialized training and connections to social services. Patients who have regular interactions with EMS and the healthcare system can also benefit from building a rapport with a consistent group of Squad 80 EMTs, who create positive, trusting relationships over time that may open the door to successful interventions and uptake of recovery and social services referrals.

There is a growing policy [1] and scholarly [2–4] discussion about how best to respond to the complex and distinct prehospital needs of vulnerable urban populations. Traditionally, EMS systems have dispatched transporting ambulances to all incident types. Today, the concept of specialized non-transporting squads in EMS is a promising operational response to calls for advancing community paramedicine; better care for addiction, homelessness, mental distress, and food insecurity; and equitable public services. To address long-standing health inequities, which the pandemic along with a nationwide discussion about health care disparities brought to the top of the urban policy agenda, cities from Austin to San Francisco to New York are segmenting services to deliver innovative forms of prehospital care.^1^ This major shift in cities' EMS policies is complemented by a landmark federal program, Emergency Triage, Treat, and Transport (ET3), which for the first time with new reimbursement structures enables at a wide-scale alternative non-transporting models of prehospital care [1].

This field study is to our knowledge the first to give an operational perspective on the emerging policy shift in urban ambulance care toward service segmentation. Segmentation is one of the most promising paths toward affirmative ambulance care, but is in some sense orthogonal to the conventional operations doctrine of pooling services. Pooled systems offer all patients standard basic and/or advanced life support, while segmented systems work to meet some patients’ specific needs with squads designed to address public health challenges like homelessness or addiction. In this practice-based study, we distill designing and evaluating Boston’s Squad 80 into two questions, which are attentive to the trade-off between segmentation and pooling. In designing Squad 80 in 2017, we ask: under what conditions is segmentation feasible, or practical? In evaluating it today, we ask: along what dimensions is the segmented squad effective? We explore this question from a patient care perspective, where among other operational concerns timeliness may be paramount, and a capacity planning perspective, where the quantities of interest are utilization and the factors that shape it.

Our empirical and applied contributions are threefold, built on 44 months of response-level Boston EMS data and multiple rounds of analysis. First, we characterize the operational signature of investigation incidents – high spatial concentration, sufficient volume, and low transport probability – that made a compelling policy case for segmentation to the City of Boston in 2017. This analysis justified allocating funding for personnel and a new non-transporting EMS sports utility vehicle for Squad 80. Second, our applied contribution is chronicling the process by which the analysis led to the deployment of the new squad. Third, we present an observational evaluation of the operational advantages and trade-offs of this segmentation, finding the specialized squad has significantly shorter response times and results in fewer non-medically-necessary transports to emergency departments than investigation incidents not involving Squad 80, leading to fewer ambulance unit-hours utilized across the system.

Specialized EMS squads, designed to respond quickly and carefully to homelessness, addiction, and other complex public health issues that have disparate racial and class impacts, are a promising step toward more equitable prehospital care. In 2017, Boston was an early mover in adopting a segmented EMS service to serve patients with complex housing and substance-use disorder needs, which now makes possible a retrospective analysis that offers urgent evidence to other cities currently exploring segmentation. Lessons from Boston EMS readily translate to other American urban ambulance systems experimenting with segmentation. For research, this work advances the modern operations understanding of pre-hospital care, clarifying variables and policy concerns that are central to efforts to segment service. For practice, this field study is meant to be an accessible guide to analysts and policy makers considering and evaluating new specialized service squads.

## Literature and policy questions

Building on existing operations analysis of pre-hospital care, we identify variables and empirical insights that structure the salient aspects of EMS capacity-management decisions. Then, relying on work on service segmentation in general as well as clinically-oriented healthcare research on specialized squads, we build support for the nine sub-questions that organize our pre- and post-intervention deployment analyses.

### Ambulance operations management literature

In general, a classic priority in ambulance system research and practice is managing capacity in order to deliver proficient patient care, for which appropriate on-scene arrival times is essential. Prescriptive operations work addresses the question of where to allocate ambulances to ensure timely ambulance arrivals, generally assuming a pooled service system, where all patients receive standard advanced and/or basic life support pre-hospital services. One foundational approach minimizes the number of ambulances required to obtain an acceptable service level across a jurisdiction [5]. Another foundational approach maximizes the demand that is coverable when the number of ambulances is constrained [6]. Since demand and the locations of ambulances vary through the day, a next step is to develop policies to re-locate ambulances [7]. Recent work focuses on mixing and extending these location and re-location models [8]. Another framework to study the location challenge is to treat the set of ambulances as a spatially distributed queuing system [9]. We built on the key variables – time to arrival-on-scene, uptime – that these location studies have honed, though our empirical analysis differs in investigating outcomes in the context where a portion of capacity is not pooled.

A more recent priority in research and practice is to use fine-grained demand data to empirically characterize utilization patterns, which have practical implications from dispatching and staffing ambulances to managing bed availability in hospitals. A crew that has worked a critical incident, such as a severe accident, has slower turnaround time on subsequent calls [10]. New (versus old) pairings of personnel on ambulance crews affect the speed with which a crew completes parts of calls [11]. Looking downstream, rising EMS encounters with COVID-19-positive patients predicts seven days later higher intensive-care-unit bed utilization [12]. A core result of this empirical work is that healthcare system utilization is understandably heterogenous, a finding to which we contribute with this research.

### Pre-deployment policy questions

Operations research suggests that for healthcare delivery systems, and for service and production systems more generally, leaving products and teams undifferentiated allows systems to quickly and efficiently meet a range of patient needs [13–15]. For example, only in some contexts is it efficient to reserve capacity in a primary healthcare practice for patients with urgent needs [16] or to delegate work to support staff [17]. Emergency medical services systems are usually staffed to reflect this principle, with personnel trained as generalists and differentiated only in terms of delivering basic life support or advanced life support for more critical calls. In general, ambulance systems pool this largely-undifferentiated capacity to be able to respond swiftly to the full range of incidents, with response times being a consistent area of interest in health-care operations work [18, 19]. Another reason service models are undifferentiated is that conventional EMS reimbursement policies are tied to completing transports to the hospital, which incentivizes models built around transporting patients regardless of whether that is the most beneficial treatment [20].

The first practical gap we help address in the healthcare operations literature is: what are some conditions under which segmenting an EMS service is appropriate? From a practice perspective, this is asking: what factors can analysts consider in building a policy case for EMS and city leadership? This part of our field study is the first empirical analysis that we are aware of that chronicles an analytics-driven and implemented policy shift toward EMS service segmentation in support of a vulnerable patient population.

We present the three questions investigated in 2017 in building the policy case for Boston EMS’s specialized-squad service model. The field-based understanding of frontline responders and leadership was mapped to these three questions. Appendix 1.1 formally states these questions.

- *Question Pre-1. Do investigation incidents (in the impacted area where Squad 80 would work) occur with sufficient frequency to justify introducing a dedicated EMS unit?* One common industry rule of thumb for volume management is a service needs one ambulance operational per 0.4–0.6 incidents per hour and busy systems with longer shifts may target even lower utilization (e.g., 0.3–0.5) to help manage fatigue [21]. The level of investigation-incident demand is important because without enough demand the city would be adding unneeded, dedicated capacity to the system. In general, 0.4 incidents per hour, and up to 0.6 incidents per hour, balances a truck being busy enough to be sufficiently utilized but available enough to rapidly respond when unoccupied.^2^
- *Question Pre-2: Are investigation incidents geographically concentrated?* If a substantial portion of these investigation incidents are geographically proximate, a specialized EMS unit could likely cover one area in order to maintain appropriate response times while still handling sufficient volume*.* Response times are often one criterion of high-quality care, especially when it is not clear whether or not the need is emergent, as is often the case with investigation incidents.
- *Question Pre-3: Do investigation incidents lead to fewer transports to hospitals, as compared to all other types of incidents*? This outcome is important because it indicates alternative types of needs, such as social services referrals, may be desirable to this patient population. By extension it also indicates the appropriateness of a specialized squad predicated on responding to incidents with a low likelihood of transport and, in doing so, absorbing an important and consuming segment of the demand mix. This question practically sheds light on whether the specialized squad should use a conventional ambulance or a lower-profile SUV, a core service design decision.

### Post-deployment policy questions

Recent healthcare research shows there can be advantages to giving EMS squads additional specialized training, equipment, and discretion. Specialization can, where state regulations permit, enable treatment-in-place on scene, allow transportation to appropriate facilities other than hospital emergency departments, and connect patients with appropriate social services—all of which may both improve patient care and lead to reductions in demand on EMS resources and emergency departments [2, 3]. In particular, given the nuances of these investigation incidents, there are clinical benefits of specifically training a squad to respond to them, such as in Philadelphia where a specialized EMS squad makes social services referrals [4].

The second practical gap in the healthcare operations literature that we address is: what are some operational advantages and trade-offs of a segmented service? We explore these questions from patient care and the capacity management lenses. For these patients, does the segmented service model focused on one geography lead to quicker arrivals on-scene—and in the event the patient requires transport, are any timeliness gains accrued with the swift arrival on scene carried through to the time-to-hospital? For the capacity planner, does a segmented service model with a Squad dedicated to non-transport interventions lead to few enough transports that any increases in unit-time for (non)transport incidents are offset? From a practice perspective, these questions are asking: what operational outcomes can analysts use to evaluate an ambulance service differentiated to better support a specific patient population? This part of our field study presents descriptive evidence, built on clear comparison groups and checked against a pre-deployment baseline, about the operational implications of segmentation.

In retrospectively evaluating the segmented service, six questions are explored. Appendix 1.2 formally states these questions.

- *Question Post-1. Do incidents to which Squad 80 responds have quicker times-to-scene than ones not involving Squad 80?* This gain may be possible because Squad 80 works in a more defined geographic area than most standard ambulances who may be re-posted or dispatched to different areas as demand and coverage evolve. Similarly, Squad 80 has greater proximity to the incident location and familiarity with routes to scenes and patients. Also, the lower profile SUV may allow Squad 80 to navigate Boston’s traffic and narrow streets more quickly. Timeliness gains are important for incidents that require emergent care—but are also valuable for incidents that (it turns out) do not, because, for these, the quicker a unit gets on-scene the sooner the unit is available again.
- *Question Post-2. Do incidents to which Squad 80 responds have longer times-to-hospital than ones not involving Squad 80?* Squad 80 itself cannot transport the patient. When a patient requires transport, does arrival to the hospital take longer when Squad 80 is on scene?
- *Question Post-3. Do incidents involving Squad 80 less frequently result in transports to emergency departments?* When patients have capacity to refuse transport and prefer not to go to the hospital, Squad 80 can rely on their training to provide not only clinically appropriate and permitted treatment on-scene but also connections to social service providers who may be better suited to meet patient needs than an emergency department. Due to Squad 80’s networks to connect an individual to social services, alternative destinations transport by a third party, such as a shelter, may be more accessible. Conversely, with a standard ambulance, the pathway to an emergency department may be more apparent to both the patient and the providers.
- *Question Post-4. Among incidents requiring only care on-scene, where the patient refuses a transport to the hospital, do those involving Squad 80 absorb fewer unit-hours than ones not involving Squad 80?* Care-on-scene incidents can entail meeting patients’ medical or other needs, like a social services referral. Unit-hours in EMS is the duration a unit spends responding to an incident. The unit-hours are equivalent to capacity utilized in operations management. A difference in unit-hours may occur because of Squad 80’s familiarity with patients as well as the geographic area.
- *Question Post-5. Among incidents that lead to a transport, do those involving Squad 80 require more unit-hours than ones without Squad 80?* Squad 80 itself cannot transport the patient. Squad 80 almost invariably arrives first on-scene, evaluates the patient, and initiates care. If the patient refuses transport, Squad 80 informs the ambulance that would have transported them and cancels their response. If the patient requests transport, a patient who would otherwise require one ambulance then results in two units on-scene when Squad 80 is involved.
- *Question Post-6. Does the expected value in unit-hours of incidents involving Squad 80 require fewer unit-hours than those without Squad 80?* Weighing the likelihood of an incident resulting in a transport, Squad 80 could net conserve unit-hours by avoiding utilization of a standard ambulance crew, even with spending more time on-scene with patients requiring more unit-time for incidents resulting in transport.

## Setting

Boston EMS is the primary municipal 9–1-1 emergency medical services provider for the City of Boston. With a total daytime service population of approximately 1.2 million people, the system provides basic and advanced life support with Emergency Medical Technicians and Paramedics staffing the 27 standard, or frontline, ambulances. The volume over the study period for these standard ambulances plus Squad 80 results in an average of 2,600 responses per week, of which 300 are categorized as investigations. Among EMS providers operating in larger US cities (> 300,000 residents), Boston EMS is in the top 20 for annual incident volume. Boston EMS had an operating budget of around $60 million per year at the time of the deployment.^3^

During the 9–1-1 call-taking process, Boston EMS employs Emergency Medical Dispatch, a standardized call taking and dispatching process. Generally, an emergency medical call results from a patient or someone nearby calling 9–1-1. In the case of investigation incidents, it is often somebody passing-by, which can mean uncertainty and few details about the patient’s status thus making arrival times important in case it is emergent. The call-taker electronically passes two pieces of information – incident type and location – to the dispatcher while adding notes into the computerized dispatch system for the responding unit to read on their console. The dispatcher has real-time visibility of all ambulances in the city and assigns an available and nearby ambulance to an incident. For investigations in the impacted area, the dispatcher’s policy is to direct Squad 80 to respond if it is available, and if it is not, they dispatch an ambulance in the area. Essentially, this policy allows Boston EMS to draw inferences about Squad 80’s outcomes relative to those of standard ambulances by comparing across like incidents.

Upon ambulance arrival on scene, EMTs and/or Paramedics assess and stabilize a patient by providing medical care and, in four out of five of all incidents, transports a patient to a hospital emergency department. The policy is to transport patients to a hospital, unless a patient is competent and capable of refusing transport and does so.^4^ Passers-by are a vital source of 9–1-1 calls. But people walking, or even driving, by do call 9–1-1 for patients who often do not require emergency medical care and decline transport to the hospital.

## Data and analysis

In this observational analysis, we use 44 months of response-level ambulance records, spanning 2016 to 2019 and centered on October 2017 when Boston EMS introduced Squad 80. A response begins when Squad 80 or one of the 27 standard ambulances is dispatched. In this timeframe, the Boston Computer Aided Dispatch system recorded 497,263 distinct, complete response records across all incident types. The dispatch of multiple EMS units to one incident occur for almost one in four incidents and one in eight investigation incidents respectively, in the pre-deployment period. To gain further insight, we combine our analysis with interviews with two of the six EMTs who work Squad 80, and two officials in leadership, in which we ask general questions about 1.) Squad 80, 2.) EMS training, 3.) the investigation-incident patient population, and 4.) implementation challenges and successes.

### Pre-deployment data and variables

In collaboration with analysts in the Boston Mayor’s Office Department of Information Technology in 2017, Boston EMS examined three key operational patterns in investigation incidents to examine the potential for adding a specialized squad that does not transport. Boston Mayor’s Office has advanced analytics capacity. This work quantified and validated the understanding of investigation incidents held by EMS frontline personnel and leadership.

In this analysis, we analyze data citywide and by zip code for investigation-incident type-codes—‘EMS investigation’, ‘request for EMS assistance’, and ‘unknown EMS’—and all other dispatch type-codes (which are how incidents are categorized in the central EMS database). Citywide there were 244,776 response records from the start of 2016 through September 2017, of which 29,939 are associated with investigation dispatch type-codes.

With these records we examine patterns in three variables. First, *frequency* is the count of the investigation incidents, in the city and within the impacted area, by hour. Second, spatial *distribution* is the share of citywide investigation incidents by zip code. Finally, *probability of transport* is the probability of an investigation or other type of incident resulting in a transport to a hospital. Appendix 1.1 formally states these variables and the questions we evaluate.

**Fig. 1:**
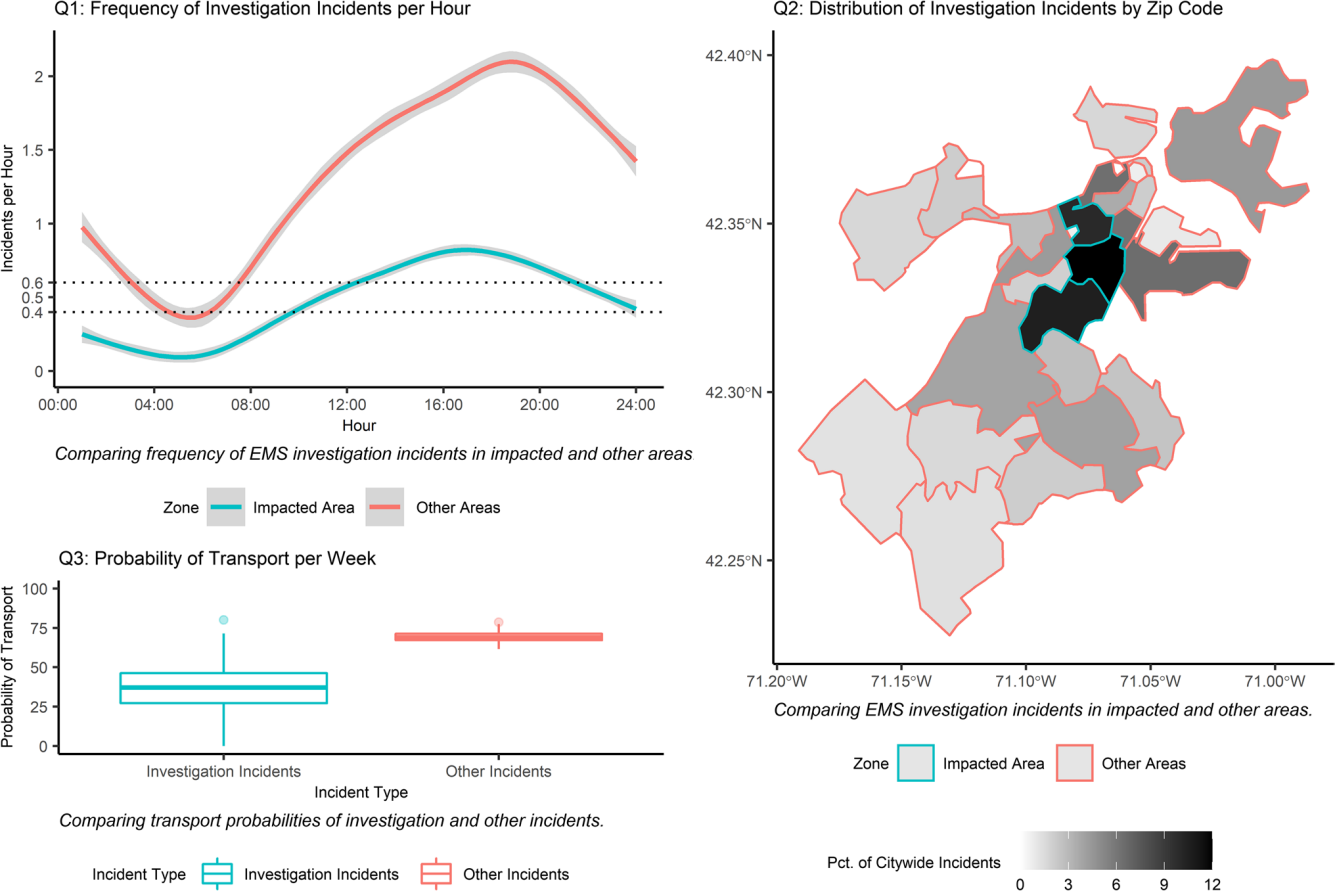
Analysis of Investigation Incidents. *Note*: Data for investigation and other incidents, by impacted and other areas, from 01–2016 to 09–2017. Results are qualitatively parallel for panels 2 and 3 if data is limited to incidents occurring during Squad 80 service hours or not. Line in panel one is of non-parametric local regression, with shading indicating the 95% confidence interval and dotted lines in this panel indicate 0.4 and 0.6 incidents per hour

In these descriptive analyses, we construct plots of each outcome variable, shared in Fig. 1.

### Post-deployment data and variables

Boston EMS’ in-house analysts compared in a retrospective, observational analysis four operational outcomes between investigation incidents involving (only) standard ambulance and those involving Squad 80. This work answered the question about the relative operational effectiveness of Squad 80.

We compare incidents in which Squad 80 was involved versus those in which it was not. Specifically, the basis of comparison is the 52 percent of investigation incidents in the impacted area when Squad 80 worked that are only handled by the standard ambulances, relative to the 48 percent of those incidents that the new Squad was involved in. Holding responding-ambulance types aside, we work with otherwise-identical incidents, because dispatch policy is to assign Squad 80 investigation incidents along with an ambulance and then standard ambulances when Squad 80 is unavailable. There is no reason to believe Squad 80 being engaged in one incident would lead to a substantively different subsequent incident, indicating ambulance assignment to these incidents is random. In Sect. 7, we carefully review patient, incident, timing, and location characteristics to ensure balance among covariates of ambulance type and detect any bias in our assessment of incidents involving Squad 80 relative to standard ambulances, due to confounding Squad 80 being assigned to an incident with a covariate.

We limit the data analyzed to incidents in the designated geographic area in the time in which Squad 80 is in service—from 08:00 am to midnight on weekdays—and to the investigation-incident dispatch type-codes to which it was created to respond. There are 5,159 response records in that area for investigation dispatch type-codes during its service hours, from October 2017 through August 2019. We summarize these records by week to average over the day-of-the-week variation typically seen in prehospital care data [22–24].

With these records we construct six outcome variables, by week and for incidents involving Squad 80 and incidents that do not. First, *time-to-scene* is the minutes between when an EMS unit is dispatched to the first unit arriving on-scene. *Time-to-hospital* is the time between the first EMS unit dispatched and the patient arriving at the hospital. P*robability of transport* is the probability an incident resulting in a patient transported to a hospital. *Unit-hours occupied* is the hours required from the responding EMS units for the entire incident response—for each, from dispatch until it is ready to respond to a new incident—also known as capacity utilized. (E.g., an incident requiring an hour each of two squads occupies two unit-hours.) We examine unit-hours occupied for *care-on-scene incidents* not resulting in a transport and for *transport incidents.* Taken together, *expected unit-time* weighs the likelihood of transport against the unit-time required for care-on-scene and transport incidents. Appendix 1.2 formally states these variables and the questions we evaluate.

This analysis is retrospective and observational. We construct plots of each outcome variable featuring a non-parametric local regression to illustrate trends across the time series. We report results from Wilcoxon rank-sum tests, a non-parametric test of independence comparable in insight to a t-test. The test makes no assumptions on the underlying distribution of the data. First, with these tests, we assess whether outcomes between incidents involving only ambulances versus those involving Squad 80 differ significantly (i.e., are drawn from distributions with significantly different medians). Second, as an additional check on Table 1, we examine if outcomes differ significantly for the ambulances before and after Squad 80 was introduced. Not detecting any change in the ambulances’ operations suggests that they continued handling similar investigation incidents after Squad 80’s introduction, and thus Squad 80 handles incidents similar to them. We expect to observe no change, because the dispatch policy essentially assigns incidents to standard ambulances based on Squad 80 unavailability.

**Table 1:** Patient, Incident, Timing, and Location Characteristics for Incidents with(out) Squad 80

Variable	Incidents		Test
	Without Squad 80	With Squad 80	
Incidents	2,286	2,092	
Patient Characteristics			
*Patient Age*			*p* = *0.904*
Age (median)	48	49	Wilcoxon (W = 149,093)
*Patient Gender*			*p* = *0.381*
Female (percent)	20.8	23.3	Chi Squared (χ2 = 0.76)
Male (percent)	79.2	76.7	
*Patient Home Status*			*p* = *0.623*
Housed from Boston (percent)	43.6	42.3	Chi Squared (χ2 = 0.94)
Housed from Out-of-Town (percent)	21.9	20.4	
Unhoused (percent)	34.5	37.2	
Incident Characteristics			
*Complaint Type*			*p* = *0.115*
Unknown Medical Event (percent)	40.3	43.2	Chi Squared (χ2 = 3.87)
Medical Investigation (percent)	57.9	55.0	
Request for Medical Assistance (percent)	1.8	1.8	
*Hospital (for transport incidents)*			*p* = *1.000*
Health Safety Net Hospital (percent)	99.9	100.0	Fisher’s Exact
Other Hospital (percent)	0.1	0.0	
Timing Characteristics			
*Time of Day*			*p* = *0.148*
Rush Hour (percent)	24.7	22.8	Chi Squared (χ2 = 2.09)
Lull (percent)	75.3	77.2	
*Day of Week*			*p* = *0.121*
Monday (percent)	17.4	18.5	Chi Squared (χ2 = 7.28)
Tuesday (percent)	19.0	18.5	
Wednesday (percent)	19.8	21.3	
Thursday (percent)	19.8	20.7	
Friday (percent)	24.1	21.0	
*Season of Year*			*p* = *0.125*
Winter (percent)	19.0	19.9	Chi Squared (χ2 = 5.73)
Spring (percent)	23.6	23.6	
Summer (percent)	33.7	35.7	
Fall (percent)	23.7	20.8	
Location Characteristics			
*Location*			*p* = *0.230*
Miles from Centroid of Impacted Area (median)	0.86	0.84	Wilcoxon (W = 2,440,620)
*Neighborhood*			*p* = *0.298*
Downtown (percent)	54.3	56.6	Chi Squared (χ2 = 3.68)
Dorchester (percent)	0.7	0.5	
Roxbury (percent)	44.7	42.5	
South Boston (percent)	0.2	0.3	

This table uses Pearson’s Chi Squared test comparison between incidents without and with Squad 80 for all covariates with the exception of: a Wilcoxon Rank Sum Test comparison for the continuously measured age and distance, and a Fisher Exact Test comparison for hospital because of few (< 5) counts in one cell. There are 2,286 incidents without Squad 80 and 2,092 with Squad 80, in the impacted area during Squad 80’s service hours. For context on data: incident, timing, and location characteristics which are automatically generated by the dispatch system, versus others that are manually coded. For context on variables: (1) the state Health Safety Net (HSN) program pays acute care hospitals for some essential health care services provided to qualified uninsured and underinsured Massachusetts residents; (2) rush hour is defined as 730am-930am and 430 pm-630 pm; (3) seasons are meteorological seasons (i.e., each is 3 months and winter starts December 1).

## Results of pre-deployment analysis

The pre-deployment analysis resulted in three main conclusions. Taken together, these built the policy-case for funding and deploying Squad 80, by quantifying the substantial and distinctive operational signature of these incidents that indicated service differentiation may be appropriate. Figure 1 represents the patterns in incidents over time and geography.

- *Investigation incidents were of sufficient volume to occupy a specialized squad* as seen in the first panel (Q1) of Fig. 1, which provides support for question pre-1. On average, about one in ten of all incidents systemwide were an investigation. Furthermore, in the geographic area in which Squad 80 would operate, there was sufficient demand to justify a dedicated unit*.* About 0.4–0.6 incidents per working-hour per ambulance is an industry rule of thumb that balances the truck being busy enough to be sufficiently utilized but available often enough to rapidly respond when unoccupied. In the impacted area, there are on average more than 0.4 investigation incidents per hour starting at 09:00 (0.45 incidents) and there is a peak of 0.88 incidents per hour at 17:00. EMS systems, including Boston’s, often consist of three shifts, with ‘day’ and ‘evening’ shifts combined covering roughly 08:00 to midnight, indicating Squad 80 should be staffed with two crews working those shifts which is also when there is expected to be demand high enough to occupy them. The temporal cadence of investigation incidents – nearly all the demand occurs in hours in which a specialized squad would in expectation maintain at least 0.4 unit-hour utilization – suggests (and implementation confirms) standard ambulances would continue to absorb a portion of volume in the impacted area.
- *Incidents were geographically concentrated in central Boston,* enabling a specialized to effectively cover this impacted area of the city as seen in the second panel (Q2) of Fig. 1, which provides support for question pre-2*.* Investigation incidents were disproportionally concentrated in an area—in which Squad 80 would be assigned—spanning 3.3 square miles (made up of three contiguous zip codes in Boston, “02,116”, “02,118”, “02,119”). More than 1 in 4 (28 percent) of investigation incidents originate in that 3.3 square mile area, out of the 48.4-square-mile city. Within the impacted area, there were two particular spots in which the incidents were most common—in part of the downtown and at the intersection of Massachusetts Avenue and Melnea Cass Boulevard. The distinctive spatial concentration of investigation incidents in this area could, even for a differentiated squad, keep response times low and avoid far drives to incidents. Additionally, focusing on a specific area meant Squad 80 would master routes, key areas, incident sites, and patients, which may translate to operational advantages as well as positive clinical outcomes.
- *Investigation incidents led to fewer transports to hospitals, as compared to all other types of incidents,* as seen in the third panel (Q3) of Fig. 1, which provides support for question pre-3. Compared to all other incidents, investigation incidents resulted in a transport about half as frequently as all other incidents. When patients are located, they often refuse transportation to the hospital because they do not have an emergent medical need and were seeking shelter or asleep outside. There were instances when some patients multiple times in a week have a passer-by call 9–1-1 and refuse transport each time an ambulance responds. Sometimes, EMTs are unable to find the investigation patients, who may have moved since a passer-by placed the call. The limited need for transports would allow the city to resource Squad 80 with a lower-profile SUV equipped to deliver the same level care as a basic life support ambulance but not for transporting.

## Policy action and implementation

The pre-deployment analysis guided the policy discussion leading to the fiscal-year 2017 in which Squad 80 was first funded. The Mayor’s Office allocated about $300,000 for the initial setup plus annual cost of Squad 80 that year. Budget support was necessary for Squad 80, because in 2017 non-transport incident costs were not typically reimbursable through insurance or Medicare/Medicaid (though the recent Medicaid non-transport-incident pilot program now allows for reimbursement in some situations). The analysis supported three areas of implementation: resourcing; staffing and training; and policy development and adherence for dispatchers and Squad 80.

First, the analysis suggested the resources Squad 80 would require. In particular it indicated a low probability of transport for investigation incidents, so Squad 80 was equipped with an SUV, instead of a transporting ambulance. It was stocked with standard supplies and equipment necessary to provide a Basic Life Support level of emergency care. Squad 80 had one cell phone, which allowed for social services to have a consistent point of contact within Boston EMS and vice versa.

Second, the analysis indicated the staffing and enhanced training from which investigation-incident patients would benefit. Investigation-incident transports were geographically concentrated, suggesting a consistent and trained set of EMTs may bring operational and clinical advantages to repeat incident scenes and patients. EMT candidates for Squad 80 had a minimum of 3 years of employment with Boston EMS, submitted an essay, and were interviewed. Selected EMTs completed a week of training. The training introduced the intent of Squad 80; reviewed the data and analysis used to justify the program; introduced the EMTs to community partners and addressed how to connect patients to social services.

In particular, a cornerstone of Squad 80 is the link it has with social services partners that work in the area and can provide immediate care for patients declining transport to a hospital. The Squad 80 EMTs maintain deep relationships with staff at leading area social service providers, including Boston Health Care for the Homeless and the nonprofit Access, Harm Reduction, Overdose Prevention, and Education (AHOPE). As a way to integrate with social services, squad members may also work in an administrative capacity, reporting on city and nonprofit activities to the Boston EMS leadership and coordinating with partner agencies. The squad would also attend community meetings. By way of the initial outreach, the intent was to explain the goal of the squad to improve the public health for vulnerable populations. In retrospect, it may have been even more advantageous for the Squad 80 EMTs to have had the opportunity to start building relationships with partners well before its deployment, rather than in the fall of 2017 and forward.

Third, the analysis informed new policy for managing investigation incidents, bringing together the insights that incidents were spatially concentrated, and transports are unlikely. Dispatch assigns Squad 80 to investigation incidents within the impacted area and a transporting ambulance. If Squad 80 arrives first, as it usually does, evaluates the patient and the competent and capable patient refuses transport, Squad 80 cancels the transporting ambulance. If Squad 80 is unavailable, dispatch assigns only a standard ambulance. Squad 80 operates within Massachusetts pre-hospital statewide treatment protocols, serving as a non-transport first-response and support unit. Following these protocols, squad members act as a resource to the patient, linking them to city or nonprofit services.

In particular, the pre-deployment analysis was helpful in rebutting scope-creep in the location and type of incidents Squad 80 responds to, which could undermine the segmented service. Initially, there were questions over flexibility in its geographic and clinical scope. Yet in revealing the highly concentrated nature of these particular incidents, the pre-deployment analysis implied operational and even clinical gains of adhering to a strict dispatch policy for Squad 80. In retrospect, the uncertainty over Squad 80 s geographic and clinical scope may have arisen because there was not enough internal and external education about the intent of Squad 80 that made clear the value of it working a clearly defined geography and incident type. The post-deployment analysis, presented next, confirms what the pre-deployment analysis implied about operational gains of Squad 80 and its scope.

## Results of post-deployment analysis

The post-deployment analysis resulted in six conclusions. Overall, the post-deployment analysis reveals that, in this context, service differentiation offers operational benefits, including significantly quicker response times and fewer transports to hospitals.

As an initial baseline check, Table 1 examines balance among the covariates in the analysis. This exercise allows us to determine if there is bias in our assessment of incidents involving Squad 80 relative to standard ambulances, due to confounding Squad 80 being assigned to an incident with a covariate such as a patient characteristic.

We detect no systematic differences between patients’ ages, genders, and housing status, the latter of which is a relevant covariate based on the policy discussion on the different needs of housed and local, housed from out-of-town, and unhoused patient sub-populations. Similarly examining incident characteristics, we observe complaint types occurring at comparable rates between incidents (not) involving Squad 80. Similarly, we report transports to state-designated Health Safety Net hospitals versus to other institutions occur at similar rates. Examining the timing and location within the impacted area of these incidents, we see incidents involving Squad 80 occur comparably to those without it during rush hour when roads are congested, at similar rates over the course of the year when factors like snow slow driving, with similar dispersion relative to the centroid of the impacted area, and with similar distribution over the neighborhoods in the impacted area. Taken together Table 1 indicates a degree of balance on patient, incident, timing, and location characteristics.

**Fig. 2:**
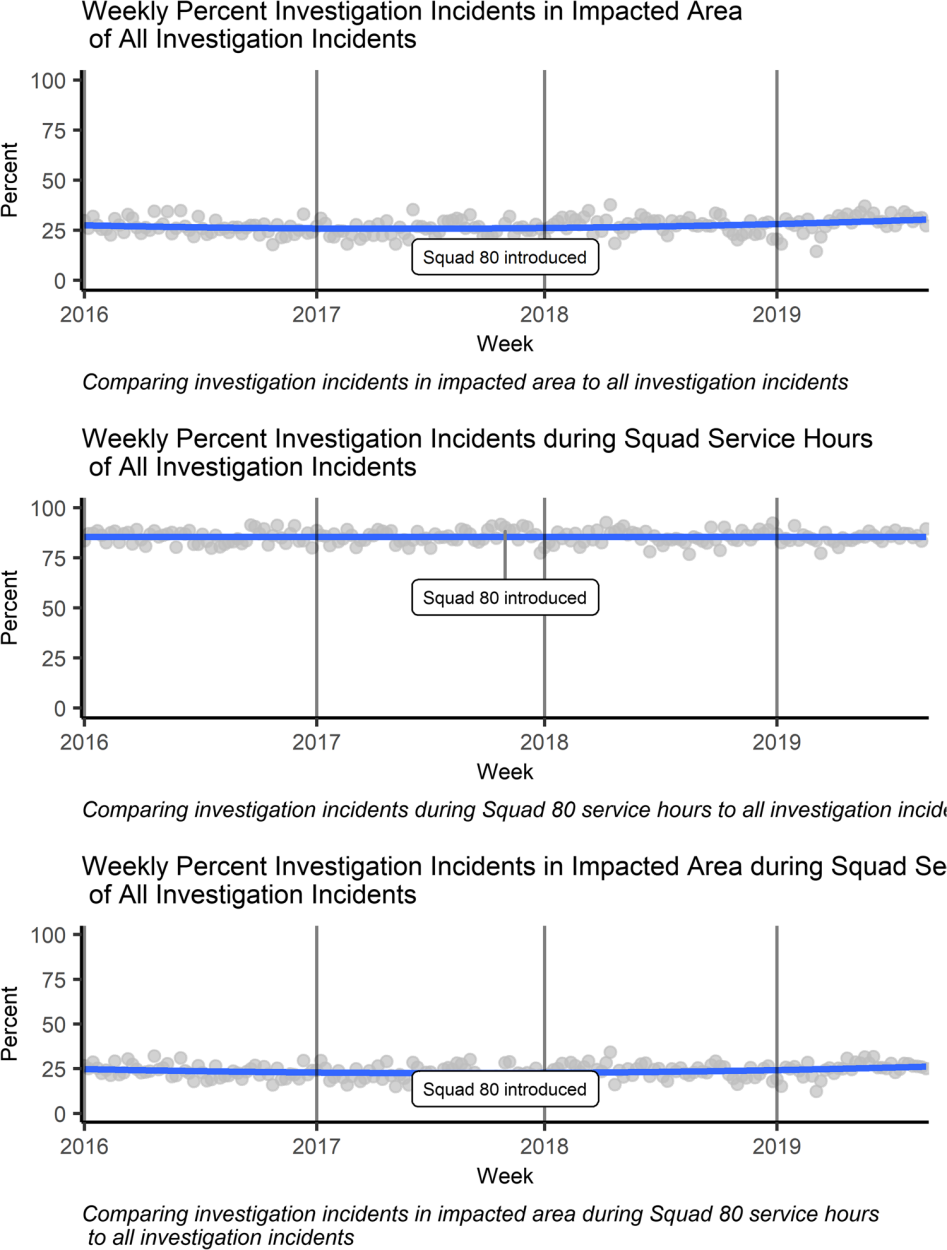
Share Investigation Incidents over Geography and Time. *Note:* Data for investigation incidents from 01–2016 to 08–2019. Lines in panels are non-parametric local regression, with shading indicating the 95% confidence interval

Next, to check the evenness of incidents over the impacted area and elsewhere before and after the deployment of Squad 80, Fig. 2 presents investigation incidents that occur in the area in which Squad 80 is assigned and the times in which Squad 80 works. The first panel shows of all investigation incidents in the system, 42 percent of them are located in the impacted area. In that area we do not detect a change in the frequency of these incidents after the deployment of Squad 80 (p = 0.356, W = 5014). The second panel shows of all investigation incidents systemwide, 80 percent occur during the hours Squad 80 is in service, and there is no detectable change in the frequency of these incidents during that window with its deployment (p = 0.677, W = 4494). Finally, the third panel shows of all investigation incidents systemwide, 34 percent occur during the hours Squad 80 works in the impacted area, and with Squad 80’s deployment we observe no statistically significant change in those incidents’ frequency (p = 0.421, W = 4468).

**Fig. 3:**
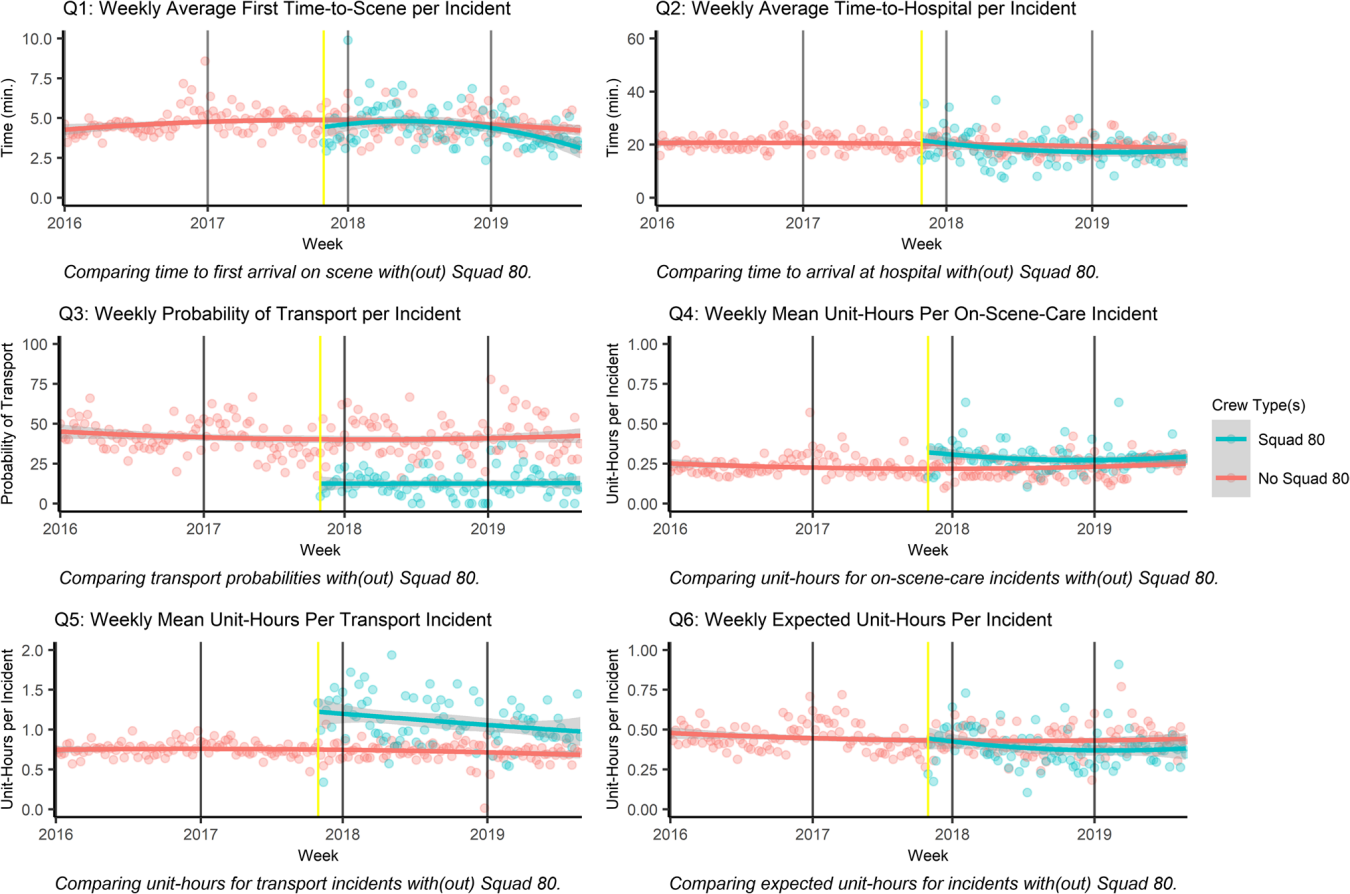
Evaluation of Squad 80 Relative to Standard Ambulances for Investigation Incidents in Impacted Area. *Note*: Data for investigation incidents in impacted area from 01–2016 to 08–2019, limited to incidents occurring during Squad 80 service hours. Yellow line indicates the deployment of Squad 80. Non-parametric local regressions of data are presented with a shaded 95% confidence interval

Next, Fig. 3 represents the operational outcomes of Squad 80 relative to standard ambulances and, as a check to ensure an appropriate comparison group, the outcomes of the ambulances before and after Squad 80’s introduction.

- *Incidents involving Squad 80 have a significantly shorter first time-to-scene than the those to which only standard ambulances respond* as seen in the first panel (Q1) of Fig. 3, which provides support for question post-1*.* Incidents with Squad 80 have a first arrival on-scene with a median of 4.09 min, compared to an arrival of 4.57 min for those without Squad 80 (p = 0.017, W = 5527, Wilcoxon rank-sum test).Shorter times may be clinically beneficial, especially given the uncertainty in the nature of the illness. To check whether this differential could result from changes in the nature or allocation of investigation incidents, we assess whether the response time for standard, Basic Life Support ambulances changes with the deployment of Squad 80. We did not detect a statistically significant difference (p = 0.472, W = 4377, Wilcoxon rank-sum test).
- *Incidents involving Squad 80 also have a significantly shorter time-to-hospital than the those to which only standard ambulances respond* as seen in the second panel (Q2) of Fig. 3*.* Incidents with Squad 80 have a time-to-hospital with a median of 17.70 min, compared to an arrival of 19.32 min for those without Squad 80 (and about 27 min for all incidents and ambulance types citywide, recognizing the impacted area is near several hospitals) (p = 0.028, W = 4907, Wilcoxon rank-sum test).Sustaining the timeliness gain accrued by a quicker time-to-scene, when a transport is required and Squad 80 arrives first, it evaluates, stabilizes, and prepares the patient for a turnover of care to the transporting ambulance crew. To test whether this differential could result from changes in the nature or allocation of investigation incidents, we assess whether the time-to-hospital for the standard ambulances changes with the deployment of Squad 80. We observe no statistically significant difference (p = 0.443, W = 4358, Wilcoxon rank-sum test).
- *The probability of an incident resulting in a transport and involving Squad 80 is significantly lower than incidents to which only standard ambulances respond* as seen in the third panel (Q3) of Fig. 3*.* This significant difference provides support for question post-3. In the median week, 12 percent of incidents to which Squad 80 responds end in a transport to the hospital, while 40 percent of comparable incidents to which only standard ambulances respond end in a transport (p < 0.001, W = 9030, Wilcoxon rank-sum test). These nearly two in three avoided transports have important implications for ambulance capacity management in the downtown part of Squad 80’s geographic area of focus. As a check, we detect no statistically significant change with the deployment of Squad 80 in the probability of transport for incidents to which only standard ambulances respond (p = 0.890, W = 4602, Wilcoxon rank-sum test).
- *The unit-hours occupied for care-on-scene incidents involving Squad 80 are significantly higher than such incidents involving only standard ambulances* as seen in the fourth panel (Q4) of Fig. 3*.* Care-on-scene incidents do not result in a transport. In observing significantly higher unit-time utilized, we do not find support for question post-4. The median time for the care-on-scene incidents involving Squad 80 is 0.28 h as compared to 0.21 h for those only involving standard ambulances (p < 0.001, W = 2339, Wilcoxon rank-sum test). The EMTs working on Squad 80 cite relationships with social service providers, trust with patients built from time on-scene, and intuition for scenes, among factors that make it effective, for instance in encouraging patients who refuse transportation to the hospital to make the most of social service alternatives. It takes time to build intuition for scenes, some which like the contours of homeless encampments changes over time, and to build and maintain relationships with the patients in the population. As a check, we observe no statistically significant change in the unit-hours for care-on-scene incidents involving only standard ambulances with the introduction of Squad 80 (p = 0.161, W = 4112, Wilcoxon rank-sum test).
- *The unit-hours occupied for transport incidents involving Squad 80 are significantly higher than such incidents involving only standard ambulances* as seen in the fifth panel (Q5) of Fig. 3*.* Transport incidents result in a trip to a hospital. This significant differential in unit-hours required provides support for question post-5. Transport incidents involving Squad 80 have a median total time from dispatch to clearing from the hospital of 1.05 h as compared to those without Squad 80 that require 0.72 h (p < 0.001, W = 786, Wilcoxon rank-sum test). This increase is to be expected as, for the one in nine incidents involving Squad 80 in which a transport is required, two units are occupied for some portion of the call instead of just one. There is a slight (but significant, estimating a simple linear regression model) downward trend with time in the unit-hours required for incidents involving Squad 80.^5^ As a check, we do not detect a statistically significant change with the deployment of Squad 80 in the unit-hours occupied for transport incidents involving only standard ambulances (p = 0.452, W = 4364, Wilcoxon rank-sum test).
- *The incidents involving Squad 80 (in expectation) require fewer unit-hours than those without Squad* 80 as seen in the final panel (Q6) of Fig. 3, offering support for question post-6*.* This insight brings together the insights from questions post-3, post-4, and post-5. Weighting the likelihood of an incident resulting in a transport, Squad 80 net conserves three minutes (p < 0.001, W = 5937), a gain made possible by transporting fewer patients despite spending more time for on-scene-care incidents and requiring more unit-time for transport incidents. As a check, we do not observe any statistically significant difference with the deployment of Squad 80 in expected unit-hours occupied for transport incidents involving only standard ambulances (p = 0.190, W = 4148, Wilcoxon rank-sum test).

## Discussion

We offer a timely operations perspective on the active discussion in practice [1] and healthcare scholarship [2–4] about how to best respond to the complex and distinct prehospital needs of vulnerable urban populations. EMS service models remain a relatively unchanged but crucial step in the urban healthcare delivery chain, historically and still today, broadly dispatch transporting ambulances to all incident types. Yet the pressing nature of caring for individuals experiencing homelessness, addiction, mental health issues, and food insecurity; the promise of community paramedicine and field-based definitive care; and the commitment in major cities to more equitable health outcomes, all require rethinking dimensions of how urban pre-hospital care is delivered.

First, this study presents the operational benefits of a squad with additional training that responds to investigation incidents. A specialized squad working in a tight geography – in this case, 3.3 square miles – can have significantly quicker arrivals, which have clinical benefits. When such a squad works an investigation incident it can also contribute to reducing unnecessary transports and emergency department visits while providing clinically appropriate on-scene care and referrals. When patients are competent and capable to refuse and do not want to go to the hospital, they may refuse transport. Squad 80’s known links with social services likely make the pathway to transport alternatives, like going to a shelter, more accessible to the patient, while a standard ambulance may make more apparent the pathway to an Emergency Department. As with any patient population [25, 26], (1) some investigation-incidents do require the patient’s transport to an emergency department (2) others do not, for lack of need or given more appropriate alternatives, and finally (3) others result in transport to an emergency department, in light of no apparent alternatives. In general, it is difficult or impossible to disentangle these groups in a quantitative sense with EMS dispatch data alone. The understanding among EMTs is that Squad 80 helps convert patients transported for lack of alternatives to patients declining transport in favor of receiving other, more beneficial services. In doing so, Squad 80 may contribute to preserving readiness and unit availability across the system for high-priority responses. Further, this may save costs to patients and emergency departments [27].

Building on our practice-based study, there is a need for research that further explores the benefits of service segmentation. Future observational and even experimental work should quantify additional system-level benefits. Under what conditions—such as time of day and population- or incident-density—does a differentiated ambulance lessen systemwide uptime to improve response times to other, high-priority calls? One line of work directly concerned with equity is studying the clinical effectiveness of segmentation, exploring outcomes ranging from addiction treatment and housing stability to mental wellbeing and food security. Linking EMS data to hospital and social services data, however, is extremely challenging in the United States because of the fragmented health care and welfare system. How do patient outcomes improve when they encounter a differentiated ambulance?

Second, this study reveals trade-offs to consider before deploying a squad for a specific patient population and geography. These compelling operational benefits entail marginally more unit-hours during care-on-scene incidents, as Squad 80 invests modestly more time on-scene with patients but then avoids time-intensive transports. These additional on-scene unit-hours may also deliver clinical benefits, by enabling EMTs to develop relationships with patients and refer them to social services that can help address treatment options or inability to access housing. A special data collection effort for the first fifteen weeks of its deployment indicated Squad 80 provided 46 referrals to recovery services; 136 referrals to homelessness services; and 37 blankets. Taken together, the net operations impact on the ambulance system of such a squad is positive: incidents involving Squad 80 require in expectation 0.38 unit-hours versus 0.42 unit-hours for comparable incidents without it, a ten percent difference.

A major opportunity for healthcare operations scholarship is to further characterize this and other trade-offs between flexibility, timeliness, and utilization. As a first step, we empirically show that investigation incidents in expectation require less uptime when a specialized squad is involved in the response, with slightly more time spent on-scene connecting with patients during non-transport incidents offset by, and resulting in, (many) fewer lengthy transports. Future work could investigate from a queuing theory perspective the second order effects of segmentation, identifying the conditions under which reduced flexibility in the system impacts uptime and timeliness, both for incidents requiring care from a specialize crew and for those requiring medical care from an undifferentiated crew.

Third, in revealing the substantial, lessened rate of transport when Squad 80 is involved in an investigation incident, this study has clear service-finance implications that are worth further investigation. The City of Boston directly funds Squad 80, because it does not transport patients itself and conventional EMS reimbursement policies for public- or private-insured patients or uninsured patients are tied to completing transports to the hospital. Reimbursement for this patient population is a particular challenge given unfixed addresses, often limited income, and inconsistent access to insurance, among other barriers to fully participating in the healthcare system. A service model that leads to fewer transports likely lessens the unreimbursed costs for the EMS system and to hospitals. Further studies may test the notion that investing in Squad 80 may be offset by fewer unreimbursed unnecessary transports and unreimbursed emergency department admissions. In other contexts, research may investigate how cost-sharing for such a squad between a public EMS system and local hospitals could operate.

Dramatically enlarging the potential of non-transporting units, the new federal Emergency Triage, Treat, and Transport (ET3) program for the first time enables a wide-scale alternative non-transporting reimbursement model for prehospital care for participating services [1]. One federal restriction of ET3 about which this research may provoke further discussion is the requirement that any participating unit still retain the capability to transport the patient—in other words, a non-transporting crew must still work out of a standard ambulance. State regulations, which are quite heterogenous, also shape how non-transporting squads are operationalized. However, there are advantages to working out of a sports utility vehicle, both in terms of cost but also the via the lower profile. The evidence from Boston indicates that from the patient perspective, in terms of time-to-scene and time-to-hospital, it may not be more helpful for specialized squads to work out of standard ambulances.

Fourth, this study suggests the value of carefully tracking and improving operational outcomes as a part of the broader change management effort required to implement new service models. The data indicates a learning effect occurred among Squad 80 and standard ambulances, with greater timeliness gains being realized week-over-week on transport incidents involving Squad 80 as it became more situated with patients and scenes and as all of the units involved better understood Squad 80’s role and benefits. There is an opportunity to study how internal education, relationships between crews, and even public health messaging to patients can strengthen the operational effectiveness of new varieties of ambulance care.

## Conclusion

This practice-based study presents the analytics that led to a substantive operational change in the health-care delivery supply chain in Boston and reports the outcomes of this new, replicable service model. The spatially concentrated, clinically complex, and pressing nature of investigation incidents indicates that new service models are needed, consistent with the impetus for community paramedicine and mobile-integrated health. With the growing interest and focus in expanding the scope of EMS practice, enhancing patient care coordination, and avoiding unnecessary transports to emergency departments, this study may serve as an accessible guide for emergency medical service decision-makers in other cities.

## Data Availability

Restrictions apply to the availability of these data, and so we do not make them public.

## Appendix 1 Formal statement of variables and tests

### Pre-deployment variables and tests

**Table:**

Index	Description
$p \in P =\{1, \dots , 9\}$	Type of call – dispatch code
	where *p* = *1* for investigation incidents
$q \in Q=\{1, 2\}$	Type of call – transport or not
	where *q* = *1* for transport incidents
$i \in I=\{1, \dots , 25\}$	Place of call – zip code
	where *i* = *1, 2, 3* for the impacted zip codes
$w \in W=\{1, \dots , 94\}$	Time – week of series
$d \in D=\{1, \dots , 7\}$	Time – day of week
$h \in H=\{1, \dots , 24\}$	Time – hour of day
Variable	Description
${x}_{pqiwdh}$	Count incidents

#### Questions

- Question pre-1: frequency
  1 $${\sum }_{p=1}^{1}{\sum }_{q=1}^{|Q|}{\sum }_{i=1}^{3}{\sum }_{w=1}^{|W|}{\sum }_{d=1}^{|D|}\frac{{x}_{pqiwdh}}{\left|W\right||D|}=\frac{{x}_{h}}{\left|W\right||D|}\ge 0.4 \forall h$$
  This inequality is represented in the first panel in Fig. 1. In addition to summarizing for $i \in \left\{1, 2, 3\right\},$ we also represent this inequality for $i \in \{1, \dots , 25\}$ in the first panel.
- Question pre-2: distribution
  2 $${\sum }_{p=1}^{1}{\sum }_{q=1}^{|Q|}{\sum }_{w=1}^{|W|}{\sum }_{d=1}^{|D|}{\sum }_{h=1}^{|H|}{x}_{pqiwdh}={argsort}_{i}$$
  This pattern is represented in the second panel of Fig. 1.
  Question pre-3: transport probability.
  Reducing the time dimension on ${x}_{pqiwdh}$ to the week level, we take ${\sum }_{d=1}^{|D|}{\sum }_{h=1}^{|H|}{x}_{pqiwdh}={x}_{pqiw}$
  3 $$\frac{\sum_{p=1}^{1}\sum_{q=1}^{1}\sum_{i=1}^{3}{x}_{pqiw} }{\sum_{p=1}^{1}\sum_{q=1}^{|Q|}\sum_{i=1}^{3}{x}_{pqiw} }\le \frac{\sum_{p=2}^{|P|}\sum_{q=1}^{1}\sum_{i=1}^{3}{x}_{pqiw} }{\sum_{p=2}^{|P|}\sum_{q=1}^{|Q|}\sum_{i=1}^{3}{x}_{pqiw} }\forall w$$

This inequality is represented in the third panel of Fig. 1.

### Post-deployment variables and tests

**Table:**

Index	Description
$p \in P =\{1, \dots , 9\}$	Type of call – dispatch code
	where *p* = *1* for investigation incidents
$q \in Q=\{1, 2\}$	Type of call – transport or not
	where *q* = *1* for transport incidents
$r \in R=\{1, 2\}$	Type of call – Squad 80 involved or not
	where *r* = *1* for incidents involving Squad 80
$i \in I=\{1, \dots , 25\}$	Place of call – zip code
	where *i* = *1, 2, 3* for the impacted zip codes
$w \in W=\{95, \dots , 188\}$	Time – week of series
$m \in M=\{1, 2, 3\}$	Step – respond, scene, and transport
	where *m* = *1* for response, *m* = *2* for scene, *m* = *3* for transport
$h \in H=\{1, \dots , 24\}$	Time – hour of day
	where h = 8, …, 24 for the operating hours
Variable	Description
${z}_{pqriwmh}$	Count uptime (hours)
${y}_{pqriwmh}$	Count incidents

#### Questions

First, we limit the count and uptime variables to investigation incidents in the impacted area during Squad 80’s operating hours,

$$\begin{array}{l}{\sum }_{p=1}^{1}{\sum }_{i=1}^{3}{\sum }_{h=8}^{24}{z}_{pqriwmh}= {z}_{qrwm}\\ {\sum }_{p=1}^{1}{\sum }_{i=1}^{3}{\sum }_{h=8}^{24}{z}_{pqriwmh}= {y}_{qrwm}\end{array}$$

- Question pre-1: time-to-scene (in minutes)
  1 $$\frac{\sum_{q=1}^{|Q|}\sum_{r=1}^{1}\sum_{m=1}^{1}{z}_{qrwm} }{\sum_{q=1}^{|Q|}\sum_{r=1}^{1}\sum_{m=1}^{1}{y}_{qrwm} }/60\le \frac{\sum_{q=1}^{\left|Q\right|}\sum_{r=|R|}^{|R|}\sum_{m=1}^{1}{z}_{qrwm} }{\sum_{q=1}^{\left|Q\right|}\sum_{r=|R|}^{|R|}\sum_{m=1}^{1}{y}_{qrwm} }/60 \forall w$$
  This inequality is represented in the first panel of Fig. 3 and is the object of the Wilcoxon rank-sum test.
- Question pre-2: time-to-hospital (in minutes)
  2 $$\frac{\sum_{q=1}^{|Q|}\sum_{r=1}^{1}\sum_{m=1}^{3}{z}_{qrwm} }{\sum_{q=1}^{|Q|}\sum_{r=1}^{1}\sum_{m=1}^{1}{y}_{qrwm} }/60\le \frac{\sum_{q=1}^{\left|Q\right|}\sum_{r=|R|}^{|R|}\sum_{m=1}^{3}{z}_{qrwm} }{\sum_{q=1}^{\left|Q\right|}\sum_{r=|R|}^{|R|}\sum_{m=1}^{1}{y}_{qrwm} }/60 \forall w$$
  This inequality is represented in the second panel of Fig. 3 and is the object of the Wilcoxon rank-sum test.
- Question post-3: transport probability
  3 $$\frac{\sum_{q=1}^{1}\sum_{r=1}^{1}\sum_{m=2}^{2}{y}_{prwm} }{\sum_{q=1}^{|Q|}\sum_{r=1}^{1}\sum_{m=2}^{2}{y}_{prwm} }\le \frac{\sum_{q=1}^{1}\sum_{r=|R|}^{|R|}\sum_{m=2}^{2}{y}_{qrwm} }{\sum_{q=1}^{|Q|}\sum_{r=|R|}^{|R|}\sum_{m=2}^{2}{y}_{qrwm} }\forall w$$
  This inequality is represented in the third panel of Fig. 3 and is the object of the Wilcoxon rank-sum test.
- Question post-4: uptime time for on-scene-care incidents
  4 $$\frac{\sum_{q=|Q|}^{|Q|}\sum_{r=1}^{1}\sum_{m=1}^{2}{z}_{qrwm} }{\sum_{q=|Q|}^{|Q|}\sum_{r=1}^{1}\sum_{m=2}^{2}{y}_{qrwm} }\le \frac{\sum_{q=|Q|}^{|Q|}\sum_{r=|R|}^{|R|}\sum_{m=1}^{2}{z}_{qrwm} }{\sum_{q=|Q|}^{|Q|}\sum_{r=|R|}^{|R|}\sum_{m=2}^{2}{y}_{qrwm} }\forall w$$
  This inequality is represented in the fourth panel of Fig. 3 and is the object of the Wilcoxon rank-sum test.
- Question post-5: uptime time for transport incidents
  5 $$\frac{\sum_{q=1}^{1}\sum_{r=1}^{1}\sum_{m=1}^{|M|}{z}_{qrwm} }{\sum_{q=1}^{1}\sum_{r=1}^{1}\sum_{m=|M|}^{|M|}{y}_{qrwm} }\le \frac{\sum_{q=1}^{1}\sum_{r=|R|}^{|R|}\sum_{m=1}^{|M|}{z}_{qrwm} }{\sum_{q=1}^{1}\sum_{r=|R|}^{|R|}\sum_{m=|M|}^{|M|}{y}_{qrwm} }\forall w$$
  This inequality is represented in the fifth panel of Fig. 3 and is the object of the Wilcoxon rank-sum test.
- Question post-6: expected uptime time
  $$\begin{array}{l}\left(1-\frac{\sum_{q=1}^{1}\sum_{r=1}^{1}\sum_{m=2}^{2}{y}_{prwm} }{\sum_{q=1}^{|Q|}\sum_{r=1}^{1}\sum_{m=2}^{2}{y}_{prwm} }\right)\left(\frac{\sum_{q=|Q|}^{|Q|}\sum_{r=1}^{1}\sum_{m=1}^{2}{z}_{qrwm} }{\sum_{q=|Q|}^{|Q|}\sum_{r=1}^{1}\sum_{m=2}^{2}{y}_{qrwm} }\right) +\\ \left(\frac{\sum_{q=1}^{1}\sum_{r=1}^{1}\sum_{m=2}^{2}{y}_{prwm} }{\sum_{q=1}^{|Q|}\sum_{r=1}^{1}\sum_{m=2}^{2}{y}_{prwm} }\right)\left(\frac{\sum_{q=1}^{1}\sum_{r=1}^{1}\sum_{m=1}^{|M|}{z}_{qrwm} }{\sum_{q=1}^{1}\sum_{r=1}^{1}\sum_{m=|M|}^{|M|}{y}_{qrwm} }\right)\le \\ \left(1-\frac{\sum_{q=1}^{1}\sum_{r=|R|}^{|R|}\sum_{m=2}^{2}{y}_{qrwm} }{\sum_{q=1}^{|Q|}\sum_{r=|R|}^{|R|}\sum_{m=2}^{2}{y}_{qrwm} }\right) \left(\frac{\sum_{q=|Q|}^{|Q|}\sum_{r=|R|}^{|R|}\sum_{m=1}^{2}{z}_{qrwm} }{\sum_{q=|Q|}^{|Q|}\sum_{r=|R|}^{|R|}\sum_{m=2}^{2}{y}_{qrwm} }\right)+\\ \left(\frac{\sum_{q=1}^{1}\sum_{r=|R|}^{|R|}\sum_{m=2}^{2}{y}_{qrwm} }{\sum_{q=1}^{|Q|}\sum_{r=|R|}^{|R|}\sum_{m=2}^{2}{y}_{qrwm} }\right)\left(\frac{\sum_{q=1}^{1}\sum_{r=|R|}^{|R|}\sum_{m=1}^{|M|}{z}_{qrwm} }{\sum_{q=1}^{1}\sum_{r=|R|}^{|R|}\sum_{m=|M|}^{|M|}{y}_{qrwm} }\right)\forall w\end{array}$$

#### Trend checks

A comparable, formal statement of the questions around the pre- and post-deployment trends is available upon request from the authors.

## Footnotes

1 Among others service models are: the Behavioral Health Emergency Assistance Response Division in New York City, the Austin-Travis County EMS Community Health Program, the Atlanta Mobile Integrated Health Advanced Practice Team, the San Francisco Community Paramedicine Team, and the southern King County (Washington) Fire Department Community Assistance, Referrals, and Education Services unit.

2 For added context, fire companies weigh availability more heavily than EMS because of the cascading effects of a slow arrival on scene, and often plan for up to 0.25, with a hard maximum of 0.3, incidents per hour, with some companies averaging as few as 0.1 incidents per hour.

3 The volume of annual responses today is 11% higher than that over the study period. The annual operating budget has also grown since 2017.

4 As in other states, Massachusetts state EMS protocols require that in order to refuse transport a patient must be legally competent (generally an adult or emancipated minor) and physically and mentally capable (have the capacity to understand the nature of the medical condition, the risks and benefits associated with the proposed treatment, and the risks associated with refusal of care) to complete an informed refusal.

5 As this is one of the most visually definitive trends, we take an additional step and estimate a simple linear regression model, where the outcome is the unit-hours occupied for transport incidents for incidents involving Squad 80 and the independent variable is week of the time series starting with the implementation of Squad 80. For one week increase in the time series, there is -0.004 (0.0014) fewer unit-hours occupied.
